# Sequential Transplantation of Haploidentical Stem Cell and Unrelated Cord Blood With Using ATG/PTCY Increases Survival of Relapsed/Refractory Hematologic Malignancies

**DOI:** 10.3389/fimmu.2021.733326

**Published:** 2021-11-04

**Authors:** Hua Li, Xiaofan Li, Yiling Chen, Duihong Li, Xianling Chen, Zhijuan Zhu, Yiting Wang, Jiafu Huang, Ping Chen, Yuanzhong Chen, Nainong Li

**Affiliations:** ^1^Hematopoietic Stem Cell Transplantation Center, Fujian Institute of Hematology, Fujian Provincial Key Laboratory on Hematology, Department of Hematology, Fujian Medical University Union Hospital, Fuzhou, China; ^2^Translational Medicine Center on Hematology, Fujian Medical University, Fuzhou, China

**Keywords:** sequential transplantation, relapsed/refractory hematologic malignancies, post-transplant cyclophosphamide, umbilical cord blood transplantation, low-dose ATG

## Abstract

Allogeneic haploidentical HSCT (haplo-HSCT) and unrelated umbilical cord blood transplantation(UCBT)are used in patients lacking HLA-identical sibling or unrelated donors. With myeloablative condition and GVHD prophylaxis of using low-dose ATG and post-transplantation cyclophosphamide (PTCY), we conducted a prospective clinical trial. Of eligible 122 patients from February 2015 to December 2019 in the study, 113 patients were involved. Forty-eight patients were in the group of sequential haplo-cord transplantation (haplo-cord HSCT), and 65 patients were in the group of single UCBT. The primary endpoint of 2-year disease-free survival (DFS) was no statistical difference between groups (64.1 *vs.* 56.5%), *p*>0.05. The analysis of subgroup patients with relapsed/refractory showed haplo-cord HSCT was associated with better OS (HR 0.348, 95% CI, 0.175–0.691; *p*=0.0025), DFS (HR 0.402, 95% CI, 0.208–0.779; *p*=0.0069), and GRFS (HR 0.235, 95% CI, 0.120–0.457, *p*<0.0001) compared to the single cord group. The 2-year’s probability in OS, DFS, and GRFS was 64.9 *vs.* 31.6%, 64.5 *vs.* 31.6%, and 60.8 *vs.* 15.0% in the haplo-cord group and single cord group, respectively. III-IV acute GVHD 8.3 *vs.* 6.2%, chronic GVHD 25.8 *vs.* 13.7%, and extensive chronic GVHD 5.3 *vs.* 1.8% were shown in corresponding group, *p*>0.05. The patients engrafted persistently with UCB showed better survival outcomes. Our sequential Haplo-cord HSCT with ATG/PTCY improved the survival of patients and might be an alternative transplantation approach for patients with relapsed/refractory hematologic malignancies.

## Introduction

Allogeneic hematopoietic stem cell transplantation (allo-HSCT) is a curative treatment for hematological malignancies, especially for patients with relapsed/refractory hematologic malignancies. The success of allo-HSCT relies on the prevention of deleterious graft-*versus*-host disease (GVHD) while sparing the beneficial graft-*versus*-leukemia (GVL) activity mediated by donor immune cells. The multicenter study of the Acute Leukemia Working Party (ALWP)/European Society for Blood and Marrow Transplantation (EBMT) showed that leukemia-free survival (LFS) and overall survival (OS) have been significantly improved in the last two decades owing to decreased non-relapse mortality (NRM). The decreasing incidence of GVHD contributed to the declining of NRM (1, 2).

For patients lacking HLA-matched donors, UCBT and haplo-HSCT can be used as an alternative (2–4). UCB as grafts has rapid availability, natural immune advantages of GVL effect, low GVHD, and low risk of disease relapse (5, 6). However, delayed hematopoiesis and immune reconstitution increased the risk of infection and early transplant-related death due to the small number of stem cells (7). Haplo-HSCT has the advantage of rapid engraftment because of the higher number of stem cells, but a higher incidence of GVHD and NRM is its disadvantage (8, 9). Recent studies showed no conclusions whether two grafts (dUCB or haplo-cord) transplantation could enhance the GVL effect along with a graft-*versus*-graft (GVG) response. The findings remain controversial (10–13). Further investigation might be explored whether two transplanted grafts of haploid-UCB could reduce or delay relapse by keeping GVL. Furthermore, multiple centers showed that the outcome of haplo-HSCT was similar to it with matched unrelated donors (14, 15). The current popular strategies for GVHD prophylaxis are Beijing protocol (GIAC) (16, 17) and post-transplantation cyclophosphamide (PTCY)-based regimens (18, 19). For improving the overall efficacy with the myeloablative condition to combine unrelated UCBT and haplo-SCT, the transplantation strategy was applied to taking advantage of the GIAC protocol having higher engraftment and lower relapse plus using PTCY for lower GVHD rate and NRM. We name the modified transplantation strategy “the competitive transplantation of haplo-cord.”

To verify the sequential transplantation strategy of haplo-cord with the integration of low-dose ATG and PTCY-based GVHD prophylaxis could increase of GVL and decrease GVHD rate, we compared the haplo-cord HSCT with single umbilical cord blood transplantation by a prospective clinical trial, which was conducted at the Hemopoietic Stem Cell Transplantation Center, Fujian Medical University Union Hospital.

## Methods

### Study Design

**Figure 1:**
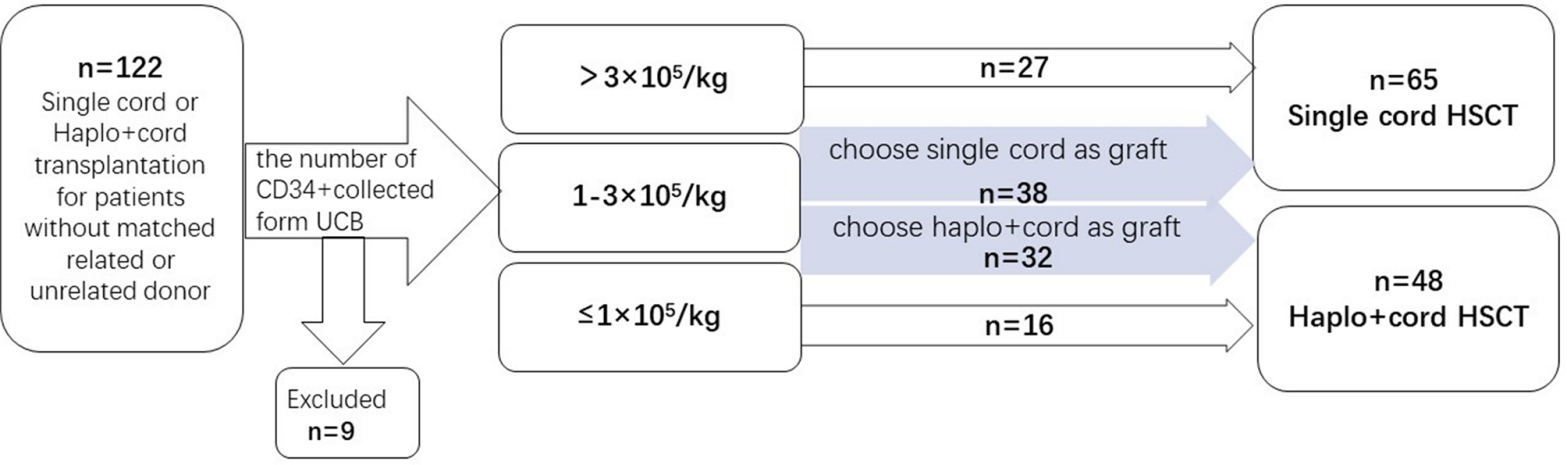
Flowchart of the cohort.

A prospective clinical trial was conducted at the Hemopoietic Stem Cell Transplantation Center, Fujian Medical University Union Hospital. Patients with no HLA matching siblings and unrelated donors were enrolled and grouped when recipient and cord blood unit HLA loci matching was ≥5/10. Then, patients were divided into different groups according to CD34^+^ cell number of UCB. The number of CD34^+^ cells was collected from UCB. If the number of CD34^+^ cells collected from a single UCB was ≥3.0×10^5^/kg of the recipient’s body weight before freezing, the patients would be assigned to the group of single cord HSCT. For UCB CD34^+^ cell number ≤1.0×10^5^/kg, the patients would be assigned to the haplo-cord HSCT group. For CD34^+^cells between 1.0 and 3.0×10^5^/kg, the patients would go to the group of single UCBT or haplo-cord HSCT group, according to patients’ willingness. Enrollment began in February 2015 and ended in December 2019. Patients were enrolled in this study if they met the following criteria: (1) Patients with hematologic malignancies in complete remission (CR) and non-remission (NR) when transplanted; (2) no matched related or unrelated donor; (3) agree to receive UCB or haploidentical plus UCB stem cells as grafts. Patients were excluded if their hematopoietic cell transplantation comorbidity index (HCT-CI) score was >3. The study protocol was approved by the ethics committee of Union Hospital, Fujian Medical University. Informed consent was obtained from both donors and recipients before the study. The engraftments, hematologic recovery, survival outcomes, and graft-*versus*-host diseases (GVHD) were evaluated for all patients after transplantation (**Figure 1**).

### Patients

Based on the information from literatures and the results from our previous work, the sample size was estimated according to the following parameters: α=0.05, β=0.8. The number of samples of the two groups was set to be equal. Ratio was 1:1. The DFS estimated at 2- year was 65% in the haplo +cord group and 40% in the single cord group. The time for enrollment was 4 years (January 2015 to December 2018), the time for following up was 2 years, and the total observation time was 72 months. The preliminary sample size estimated was 41 patients in the haplo + cord group and 40 patients in the single cord group. During follow-up, for considering possible subgroup analysis needed for patients, we extended the time of case collecting for one more year to December 2019. Of eligible 122 patients in the study, nine patients were excluded due to the following reasons: two patients died from severe infection before transplant, two patients failed to receive transplant due to organ dysfunction, three patients withdrew consents, and two patients abandoned transplant due to economic reasons. Finally, 48 patients were assigned to the haplo-cord transplant group and 65 patients to the single cord transplant group. Follow-up of patients was updated in December 2020. Patients’ baseline characteristics are shown in **Table 1**.

**Table 1:** Patient baseline characteristics of haplo-cord and single cord group.

	Unweighted population			IPTW population			Reduction of SMDa %
Characteristics	Haplo+Cord	Single Cord	*p* value	Haplo+Cord	Single Cord	*p* value	
**Total patients**	48	65		112	113		
**Median age at diagnosis, years (range)**	28.5 (6–57)	23.3 (1–49)	0.042	25.6 (6–57)	25.7 (1–49)	0.976	98.460
**Weight (range)**	62.6 (20.1–90.0)	49.3 (10.0–80.0)	0	55.1 (20.1–90.0)	54.7 (10.0–80.0)	0.890	96.660
**Median follow-up months (range)**	21.6 (1.5–49.6)	36.2 (0.1–71.9)	/	22.9 (1.5–49.6)	37.3 (0.1–71.9)	/	
**Patient gender n(%)**			0.055			0.975	
Male	31 (64.4)	31 (47.7)		71 (63.5)	62 (55.3)		
Female	17 (35.6)	34 (52.3)		41 (36.5)	51 (44.7)		
**Diagnosis n (%)**			/			/	
AML	21 (43.8)	30 (46.2)		47 (41.6)	53 (47.2)		
ALL	23 (47.9)	34 (52.3)		56 (49.7)	55 (49.3)		
MDS	3 (6.3)	0 (0)		8 (7.2)	0 (0)		
CML-AP	1 (2.0)	1 (1.5)		1 (1.5)	5 (3.5)		
**HCT-CI score n (%)**			0.701			0.593	
0–1	45 (93.7)	62 (95.4)		108 (96.4)	107 (94.7)		
2	3 (6.3)	3 (4.6)		4 (3.6)	6 (5.3)		
**Interval diagnosis to transplant n (%)**			0.956			0.876	
<12 months	33 (68.8)	45 (69.2)		75 (67.1)	76 (67.3)		
>=12 months	15 (31.2)	20 (30.8)		37 (32.9)	37 (32.7)		
**Disease status at transplant, n (%)**			0.02			0.643	84.200
First CR	20 (41.6)	42 (64.6)		54 (48.3)	61 (54.0)		
Second CR or greater	7 (14.6)	10 (15.4)		21 (19.2)	17 (15.1)		
Refractory/Relapse	21 (43.8)	13 (20.0)		37 (32.5)	35 (30.9)		
**MRD status with CR at transplant, n (%)**			0.786			0.776	
MRD positive	6 (22.2)	10 (19.6)		15 (20.7)	17 (22.6)		
MRD negative	21 (77.8)	43 (80.4)		60 (79.3)	58 (77.4)		

IPTW, inverse probability of treatment weighting; SMD, standardized mean differences.

### Donors’ Characterization and Selection

#### Haploidentical Peripheral Blood

Following the standard haploidentical criteria with high resolution of HLA-A and HLA-B, HLA-C, HLA-DR, HLA-DQ, the grafts were required to match for at least 5 of 10 HLA loci. After 10 μg/(kg/day) of G-CSF was used subcutaneously for 5 consecutive days, related haploidentical donor stem cells (from their parents, offsprings, or siblings) were collected by apheresis. The apheresis was started on day 5 of stem cell mobilization and continued daily until at least 5×10^8^/kg peripheral blood total nucleated cells (TNC) or 2×10^6^/CD34^+^ cells/recipient (kg) were collected.

#### Cord Blood

The UCB was from the Public Cord Blood Banking in China. The cord blood unit was also selected based on the high resolution of HLA-A and HLA-B, HLA-C, HLA-DR, HLA-DQ. The unit was required to match for at least 5 of 10 HLA loci. Total nucleated cells transplanted were not less than 1×10^7^/kg of the recipient’s body weight before freezing. HLA matching was prioritized over cell dose.

### Patients’ Donor-Specific Antibodies

The donor will not be included if the recipient has donor-specific antibodies against the high-expression HLA with mean fluorescence intensity >2,000.

In addition to the requirement above, HLA Compatibility, ABO match, Sex of donor to recipient, and more details are shown in **Table 2**.

**Table 2:** Graft characteristics of haplo+cord and single cord group.

Characteristics	n (%)	
Type of donor	Haplo+cord	single cord
	n=48	n=65
**Sex of Haplo-donor to recipient**		
Male to male	20 (41.7)	/
Female to male	8 (16.7)	/
Male to female	17 (35.3)	/
Female to female	3 (6.3)	/
**HLA Compatibility of Haplo-blood unit**		
5/10	30 (62.5)	/
6/10	9 (18.7)	/
7/10	6 (12.5)	/
≥8/10	3 (6.3)	/
**ABO match from Haplo-donor to recipient**		
matched	24 (50)	/
Major mismatched	13 (27)	/
Minor mismatched	8 (16.7)	/
Major and minor mismatched	3 (6.3)	/
**Sex of UCB donor to recipient**		
Male to male	19 (39.6)	21 (32.3)
Female to male	12 (25.0)	10 (15.4)
Male to female	5 (10.4)	20 (30.8)
Female to female	12 (25.0)	14 (21.5)
**HLA Compatibility of cord-blood unit**		
5/10	7 (14.6)	1 (1.6)
6/10	17 (35.4)	20 (31.2)
7/10	15 (31.3)	23 (35.9)
≥8/10	9 (18.7)	20 (31.3)
**ABO match Cord-donor to recipient**		
Matched	10 (20.8)	21 (32.3)
Major mismatched	16 (33.3)	20 (30.8)
Minor mismatched	17 (35.4)	10 (15.4)
Major and minor mismatched	5 (10.4)	14 (21.5)
**Number of Graft** (Mean ± SEM)		
Cord TNC ×10^7^/kg	2.55 ± 1.87	5.49 ± 4.25
Cord CD34^+^cells, ×10^5^/kg	1.21 ± 0.58	4.12 ± 3.58
Haplo TNC×10^8^/kg	16.28± 13.19	/
Haplo CD34^+^cells, ×10^6^/kg	7.33 ± 4.71	/

### Preparative Regimen

**Figure 2:**
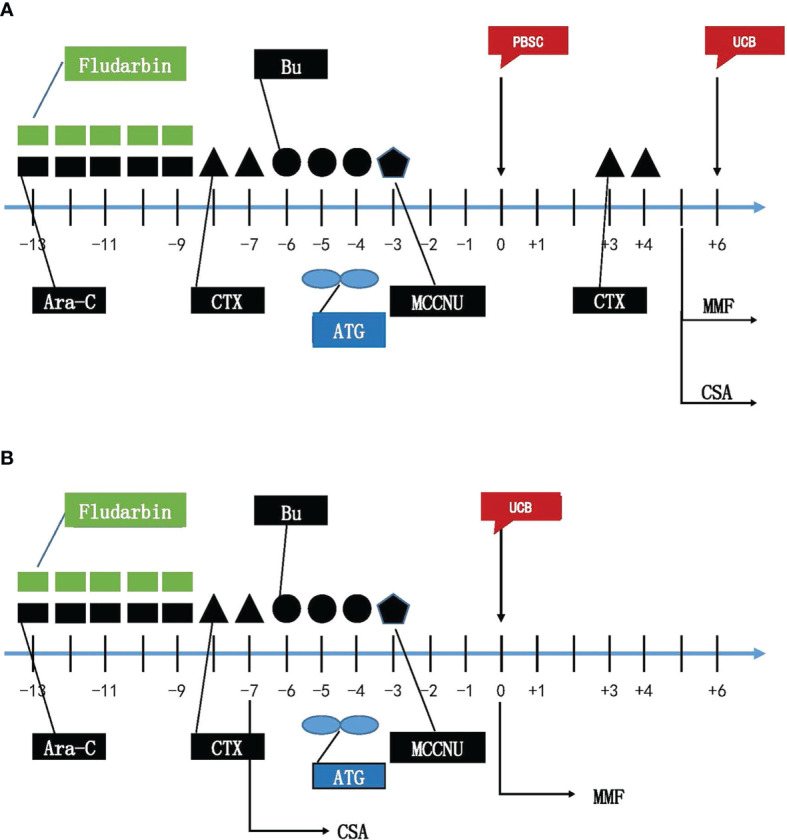
Myeloablative Conditioning Regimens for haplo-cord HSCT **(A)** and single cord HSCT **(B)**. Flu, Fludarabine; Ara-C, cytarabine; CTX, Cyclophosphamide; Bu, busulfan; ATG, anti-thymocyte globulin; MMF, Mycophenolate mofetil; CsA Cyclosporin; PBSC, peripheral blood hematopoietic stem cells; UCB, umbilical cord blood stem cells.

Myeloablative condition (MAC) regimen for all recipients was as follows: (1) Fludarabine (25 mg/m^2^, iv, daily), Cytarabine (Ara-C, 2 g/m^2^, iv, daily) from day −13 through −9; (2) Cyclophosphamide (Cy, 1.8 g/m^2^, iv, daily) on day −8 and day −7, Mesna (2.5 g/m^2^) was given for salvage to prevent early bladder hemorrhage induced by Cyclophosphamide; (3) Busulfan (Bu, 0.8 mg/kg, iv, q6h) from day −6 through −4, and Phenytoin sodium was given to prevent seizure induced by Busulfan; (4) MCCNU (250 mg/m^2^, PO) on day −3 to prevent central nervous system leukemia (CNSL). Peripheral stem cells were transfused on day 0, and umbilical cord blood stem cells were transfused on day 6 in haplo-cord HSCT (**Figure 2A**). UCB stem cells were transfused on day 0 in single cord HSCT (**Figure 2B**).

### GVHD Prophylaxis

Patients in the haplo-cord transplantation group received low dose of anti-thymocyte globulin (ATG), PTCY, cyclosporine A (CSA), and mycophenolate mofetil (MMF): (1) ATG (total dose 5 mg/kg, iv) on day −5 and −4; (2) Cy (1.8 g/m^2^, iv) on day +3, +4; (3) from day +5, CsA (3.0 mg/kg/day, iv) was provided until stem cell engrafted. Then, CsA was taken orally, targeting the blood concentration range of 200–300 ng/ml for 3 months. CSA dose was reduced to 5–10% every week for 6 months and then fully stopped. If the patient is intolerant to CsA, Tacrolimus is adjusted to maintain a concentration of 5–15 ng/ml till day 180. (4) MMF (1.0 g/day) was provided from day +5 to stem cell engrafted. Then, MMF was reduced to 0.5 g/day for 1 month and stopped. For patients who received single cord HSCT, all patients received a low dose of ATG, CsA, and MMF: (1) ATG (total dose 5 mg/kg, iv) on days −5 and −4. (2) From day −7, CsA (3.0 mg/kg/day, iv) was provided until stem cell engrafted. Then, oral CsA was administered, CsA blood level and dosage reduction were consistent with haplo-cord group. (3) MMF (1.0 g/day) was provided after UCB infusion on the day +0 to stem cell engrafted, then MMF was reduced to 0.5 g/day for 1 month and fully stopped. If GVHD level was over grade II in the two groups, glucocorticoid, anti-CD25 antibody, FK506, and anti-TNFα antibody were provided for GVHD treatment.

### Infectious Prophylaxis

Oral gentamicin and nystatin were given to treat intestinal bacteria infection. Cotrimoxazole (SMZ) was for the prevention of *Pneumocystis carinii* infection; Acyclovir was given to prevent viral infections. Patients without a history of invasive fungal infection (IFI) before transplantation were treated with posaconazole to prevent fungal infection for 30–90 days after transplantation, and the patients with a history of IFI were continually treated with effective antifungal agents.

### Study Endpoints

The primary endpoint was disease-free survival (DFS), which was defined as the time to relapse, progression, or death from any cause after hematopoietic stem cell infusion. Secondary endpoints included the main post-transplant outcomes: hematologic recovery, overall survival (OS), GVHD-free, relapse-free survival (GRFS), relapse mortality (RM), non-relapse mortality (NRM), the incidence of relapse (RI), and incidence and grading of acute and chronic GVHD. Hematologic recovery was defined as when the absolute neutrophil count (ANC) was >0.5×10^9^/L for the consecutive 3 days, the myeloid was engrafted. Platelet was engrafted when platelet count was >20×10^9^/L without transfusion support for 7 consecutive days. OS was defined as the time from allo-HSCT to death, regardless of the cause. GRFS was defined as being alive neither grade III-IV aGVHD nor severe cGVHD, and without relapse at any time point. Disease relapse was defined as disease progression from the best response. Relapse was diagnosed based on clinical and pathologic criteria. Death without disease progression was considered transplantation-related. Death without evidence of relapse defined NRM. Diagnosis and grading for GVHD were performed according to published criteria (20, 21). Performance status was graded based on HCT-CI score.

### Statistical Analysis

Given this trial was an observational study lacking random assignment, there may be differences in baseline characteristics. For the following further statistical analysis, inverse probability of treatment weighted (IPTW) was used to balance the difference between groups. IPTW is an important causal inference method widely used in current observational studies, which is introduced to reduce or eliminate the effects of confounding when using observational data to estimate treatment effects (22). This method has considered confounding issues such as non-random program participation and differences in observed characteristics of subjects and further helps to reduce bias.

The main clinical and hematological variables related to patients and graft characteristics between two groups were compared using a chi-square test for categorical variables and T-tests for continuous variables. All tests of significance were two-tailed, and *p ≤* 0.05 was considered significant. Before survival analysis, age, weight, and pre-transplant status between the two groups were balanced by IPTW. After IPTW, Kaplan–Meier method was performed to estimate the probabilities of OS, DFS, and GRFS with log-rank test. All probabilities were given at 2 years and provided with their 95% confidence interval. *p*<0.05 was considered to indicate a statistically significant difference. Hazard ratio was calculated by Cox regression model used for comparison of survival outcomes (OS, DFS, GRFS, RI, RM, and NRM) between the two treatment arms. The cumulative incidences of engraftment, GVHD, relapse, RM, and NRM were calculated by competing-risks analysis. Statistical analysis was performed using SAS software(version 9.3)and R software based on the survival (version 3.2-10) and survminer (version 0.4.9) package.

## Results

### Clinical Characteristics

From February of 2015 to December of 2019, 122 patients with hematologic malignancies were enrolled in the trial (nine patients were excluded): 48 were assigned to the haplo-cord HSCT group and 65 to the single cord HSCT group. As shown in **Table 1**, the two groups were well balanced with respect to sex, HCT-CI score, the interval diagnosis to transplant, hematologic status at transplant, and MRD status with CR at transplant. Most patients with CR were MRD negative (77.8% in haplo-cord HSCT group and 80.4% in single cord HSCT group). The patients’ median age was 28.5 years (range 6–57) and 23.3 years (range 1–49) in haplo-cord HSCT group and single cord HSCT group, respectively. The patients’ median age in haplo-cord HSCT group was older than that of patients in the single cord HSCT group, and the body weight in haplo-HSCT group was heavier than the single cord HSCT group as well. Patients with relapse and refractory in haplo-cord HSCT group were 43.8%, and the single cord HST group was 20% (**Table 1**). Patient characteristics after propensity score weighting are also shown in **Table 1**. After propensity score weighting, the variables were well balanced between the two groups. Patient characteristics after IPTW are also shown in **Table 1**; the variables (age, weight and disease status at transplant) were well balanced between the two groups. The percentage of overall standardized mean differences (SMD) had dropped by 93.88%. The distribution of the estimated propensity scores in haplo-cord and single cord group is shown in **Supplementary Figure S1**. Cumulative distribution of IPTW score weights in two groups is shown in **Supplementary Figure S2**. The standardized mean differences were attenuated after IPTW (**Supplementary Figure S3**).

### Graft Characteristics, Engraftment, and Chimerism

**Figure 3:**
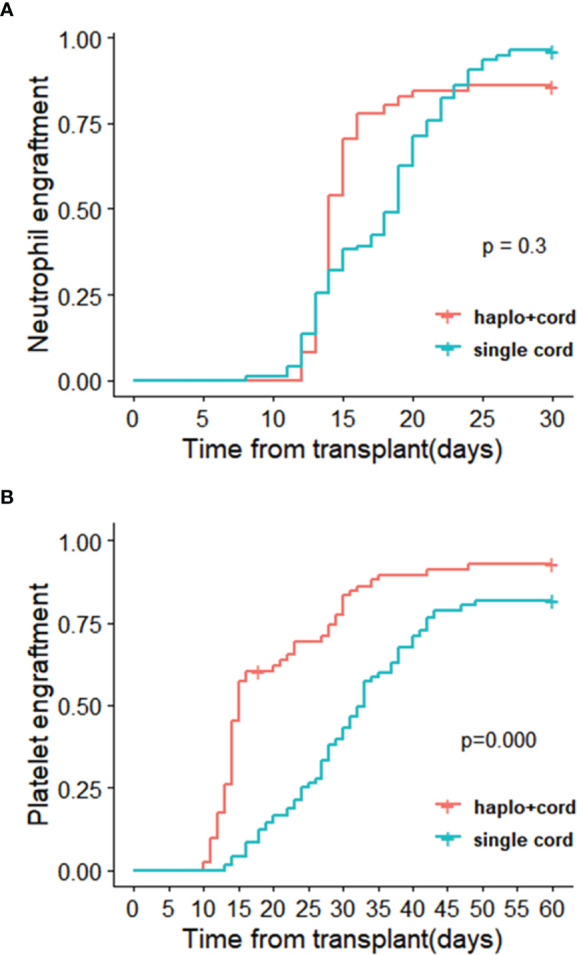
Cumulative incidences of **(A)** neutrophil recovery and **(B)** platelet recovery in haplo-cord HSCT and single cord HSCT group after IPTW.

Graft characteristics going on transplantation are described in **Table 2**. As planned, all transplanted UCB units were at least 5/10 HLA identical to the recipient. All haplo-cord recipients received haploidentical peripheral blood stem cells and a single cord blood unit. In haplo-cord group, for the cell number of TNC and CD34^+^ transplanted, (16.28± 13.19) ×10^8^/kg and (7.33 ± 4.71) ×10^6^/kg from the haploidentical donor, respectively; (2.55 ± 1.87) ×10^7^/kg and (1.21 ± 0.58) ×10^5^/kg from the cord blood donor, respectively. In the single cord group, the corresponding cell count was (5.49 ± 4.25) ×10^7^ and (4.12 ± 3.58) ×10^5^/kg from cord donor, respectively. The proportion of patients achieving neutrophil engraftment at 28 days was 85.9% (95% CI: 63.1–94.6) and 98.4% (96.2% CI: 88.4–98.8%) in the haplo-cord HSCT and single cord HSCT groups, respectively (*p*= 0.30). The neutrophil engraftments occurred at a median of 14 days (range 12–37) and 17 days (range 8–27) for the haplo-cord HSCT and single cord HSCT recipients (*p*=0.793). The 60-day cumulative incidence of platelet engraftment was 92.8% (95% CI 82.0–97.2%) and 81.7% (95% CI 66.8–90%) in the haplo-cord HSCT group and single UCB HSCT group, respectively (*p*=0.000). The corresponding platelet engraftment occurred at a median of 17 days (range 10–60) and 28 days (range 13–66), respectively (*p*=0.0004) (**Figure 3**). Recipients in haplo-cord group had engraftment of 28 (58.3%) from the haploidentical donor and 20 (41.7%) from the UCB donor. All patients achieved complete and stable chimerism (>95%) within 1–3 months after the mixed chimerism of haploidentical and cord blood cells with variable percentages. The recipients in a single UCB HSCT group had achieved 100% UCB graft chimerism within 14–21 days.

### Overall Survival Outcomes

Univariate analysis of survival outcomes was performed with the log‐rank test in overall survival outcomes. With a median time of following-up patients for 21.6 (1.5–49.6) months and 36.2 (0.1–71.9) months, there were similar outcomes after IPTW in the 2-year probability of OS (67.3 *vs.* 61.6%), DFS (65.2 *vs.* 56.6%), and GRFS (61.8 *vs.* 49.1%) in the haplo-cord HSCT group and single cord HSCT group, respectively (*p*>0.05). The corresponding 2-year cumulative incidence of relapse (11.7 *vs.* 22.8%), RM (9.6 *vs.* 16.2%), and NRM (25.6 *vs.* 26.5%) also had no statistical difference between the haplo-cord HSCT group and single cord HSCT (**Supplementary Data, Table S1**). The main causes of death were infection (40.0 and 38.1%) and relapse (26.6 and 38.1%) in the haplo-cord HSCT group and single cord HSCT (**Supplementary Data, Table S2**).

### Survival Outcomes in Complete Remission and Relapsed/Refractory Patients

**Figure 4:**
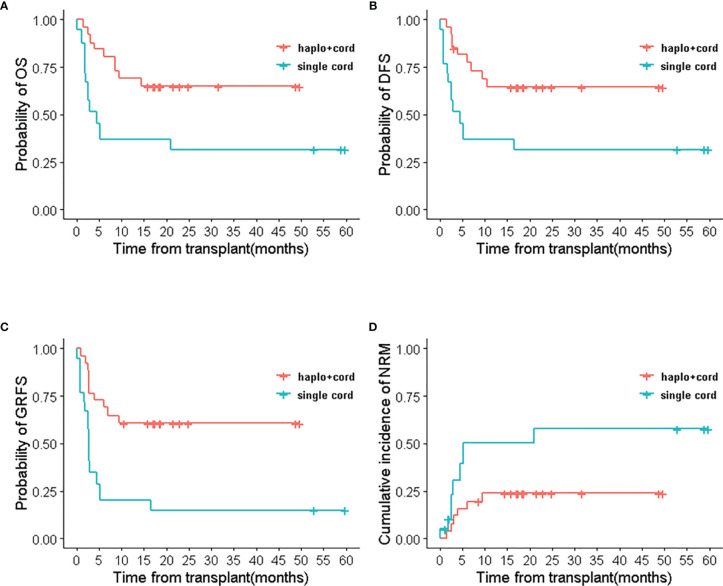
The relapse/refractory patients’ outcomes of haplo-cord HSCT and single UCB-HSCT group after IPTW. **(A)** OS, **(B)** DFS, **(C)** GRFS, **(D)** NRM.

Comparisons between subgroups were done for patients with complete remission and relapsed/refractory in two groups. In the multifactor Cox regression analysis, we made a model with the treatment regimen, pre-transplant status, and their interaction. The results indicated that the group (treatment regimen) and the interaction between groups and pre-transplantation status did not affect RM (*p*> 0.05), but the pre-transplant status had a significant impact on RM (*p*<0.05). The interactions were statistically significant in OS, DFS, GRFS, relapse, and NRM (*p* < 0.05). This indicated that the treatment regimen for the pre-transplant status with different stratification caused the different survival outcomes (**Supplementary Data, Table S3**). Therefore, survival analysis with the Cox regression model was performed for pre-transplant states with different stratifications. Results showed that treatment regimen could not change the survival outcomes in stratifications with CR1 and ≥CR2 (including MRD-positive or MRD-negative) (*p*>0.05) (see **Table 3** and **Supplementary Data, Table S4**). But the analysis for NR with stratification, the treatment regimen had an obvious impact on the outcomes. Haplo-cord group was associated with better OS (HR 0.348, 95% CI, 0.175–0.691; p=0.0025), DFS (HR 0.402, 95% CI, 0.208–0.779; p=0.0069), and GRFS (HR 0.235, 95% CI, 0.120–0.457, *p*<0.0001) compared to the single cord group. The data showed the superior outcomes of 2 year’s probability in OS 64.9% (95% CI 41.6–88.2%), DFS 64.5% (95% CI 40.7–88.3%), and GRFS 60.8% (95% CI 37.0–84.6%) in the haplo-cord HSCT group compared to that of 31.6% (95% CI 2.3–60.8%), 31.6% (95% CI 2.4–60.7%), and 15.0% (95% CI 0–34.4%) in the single cord HSCT group. Multivariate analysis failed to show significant differences in Relapse, RM, and NRM between the groups. The corresponding 2-year cumulative incidences of relapse, NRM, and RM were 18.7% (95% CI 0–39.9%) *vs.* 35.4% (95% CI 0–71.9%), 23.8 (95% CI 3.0–44.7%) *vs.* 57.8 (95% CI 2.9–93.6%), and 14.7 (95% CI 0–34.5%) *vs.* 25.2 (95% CI 0–52.7%) in the haplo-cord and single cord HSCT group, respectively. HR and their 95% confidence interval are shown in **Table 3**; Kaplan-Meier survival curves are shown in **Figure 4**.

**Table 3:** Cox regression analysis for different pre-transplant disease status between two groups.

			OS		DFS		GRFS		RI		NRM		RM	
			HR (95%CI)	*p*	HR (95%CI)	*p*	HR (95%CI)	*p*	HR (95%CI)	*P*	HR (95%CI)	*p*	HR (95%CI)	*p*
**unweighted**	**CR1**	Single cord												
		Haplo+cord	1.249	0.7227	0.953	0.9295	1.069	0.8933	0.806	0.7902	1.311	0.7074	1.104	0.937
			(0.365, 4.27)		(0.331, 2.744)		(0.401, 2.85)		(0.164, 3.961)		(0.318, 5.399)		(0.095, 12.879)	
	**≥CR2**	Single cord												
		Haplo+cord	0.989	0.9876	0.989	0.9876	0.768	0.7098	NA	NA	1.492	0.6716	0.517	0.5646
			(0.235, 4.152)		(0.235, 4.152)		(0.192, 3.078)				(0.235, 9.493)		(0.055, 4.881)	
	**NR**	Single cord												
		Haplo+cord	0.310	0.0148	0.311	0.0146	0.303	0.0087	0.534	0.4295	0.457	0.1436	0.357	0.2382
			(0.121, 0.795)		(0.122, 0.794)		(0.124, 0.739)		(0.113, 2.531)		(0.16, 1.305)		(0.064, 1.977)	
**weighted**	**CR1**	Single cord												
		Haplo+cord	1.072	0.8784	0.841	0.6513	1.059	0.8676	0.651	0.4571	1.255	0.6622	0.696	0.7012
			(0.441, 2.609)		(0.398, 1.779)		(0.537, 2.089)		(0.21, 2.019)		(0.454, 3.469)		(0.11, 4.421)	
	**≥CR2**	Single cord												
		Haplo+cord	1.614	0.2666	1.614	0.2667	1.413	0.4061	NA	NA	2.456	0.0633	0.294	0.1779
			(0.694, 3.757)		(0.693, 3.757)		(0.625, 3.192)				(0.952, 6.342)		(0.05, 1.744)	
	**NR**	Single cord												
		Haplo+cord	0.348	0.0025	0.402	0.0069	0.235	<.0001	0.595	0.321	0.463	0.0599	0.442	0.15
			(0.175, 0.691)		(0.208, 0.779)		(0.120, 0.457)		(0.214, 1.658)		(0.207, 1.033)		(0.145, 1.344)	

NA, Not Available.

### GVHD

No statistical significance was obtained in the 2-year cumulative incidences of Grade III-IV acute GVHD (aGVHD), cGVHD, and extensive cGVHD between the two groups.

The 2-year cumulative incidences of grade II-IV acute GVHD in the haplo-cord HSCT group were higher than that of the single UCB HSCT group. The incidence of grade II-IV aGVHD was 35.4% (95% CI 23.7–50.6%) in the haplo-cord HSCT group and 12.3% (95% CI 6.4–23.1%) in the single UCB HSCT group. The corresponding incidences of grade III-IV acute GVHD were 8.3% (95% CI 3.2–20.7%) and 6.2% (95% CI 2.4–15.6%), respectively, *p*=0.635. The 2-year incidences of chronic GVHD were also similar in the two groups, 25.8% (95% CI 14.8–42.7%) and 13.7% (95% CI 6.7–26.7%) after haplo-cord blood and single UCB transplantation, respectively (*p*=0.135). The severity of chronic GVHD did not differ between the two groups. The incidence of extensive chronic GVHD was 5.3% (95% CI 1.4–19.7%) in the haplo-cord HSCT group and 1.8% (95% CI 0.3–12.2%) in the single UCB HSCT group (*p*=0.391) (**Table 4**).

**Table 4:** Cumulative incidence of GVHD between groups.

	Cord-haplo	Single Cord	p
	% (95% CI)	% (95% CI)	
**100-day aGVHD**			
II -IV	35.4 (23.7–50.6)	12.3 (6.4–23.1)	0.003
III-IV	8.3 (3.2–20.7)	6.2 (2.4–15.6)	0.635
**2-year cGVHD**			
cGVHD	25.8 (14.8–42.7)	13.7 (6.7–26.7)	0.135
extensive cGVHD	5.3 (1.4–19.7)	1.8 (0.3–12.2)	0.391

### Infections

Patients in the single cord group have significantly higher rates of septicemia (*p*<0.05). CMV infection and EBV viremia had no statistically significant differences between the two groups (*p*>0.05) (**Table 5**).

**Table 5:** Septicemia, CMV, and EB infection after transplantation in two groups.

	Haplo+Cord	Single Cord	*p*
Diagnosis (n, %)			
Septicemia	9 (18.8)	30 (46.2)	0.0025
CMV viremia	39 (81.3)	60 (92.3)	0.078
EB viremia	20 (41.7)	26 (40.0)	0.859

### Survival Outcomes According to Different Engraftment Types in Haplo-Cord HSCT

**Figure 5:**
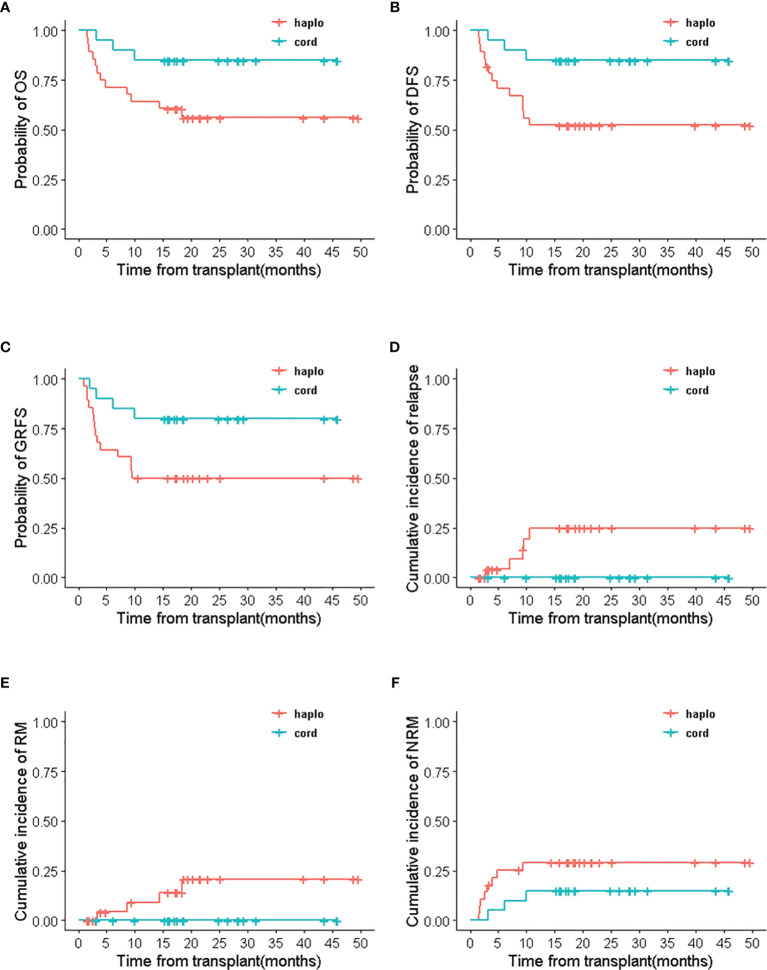
The survival outcomes of haploidentical engraftment and umbilical cord blood engraftment in haplo-cord HSCT group. **(A)** OS, **(B)** DFS, **(C)** GRFS, **(D)** RI, **(E)** RM, **(F)** NRM.

Survival outcomes of recipients were related to the types of engraftments in haplo-cord HSCT group. Patients with umbilical cord blood engraftment in haplo-cord group had better survival outcomes than recipients with haploidentical engraftment. The 2-year OS, DFS, and GRFS were all 85.0% (95% CI 70.7–100%) in patients with UCB engraftment and 56% (95% CI 40.0–78.5%), 50 %(95% CI 34.5–72.4%), and 50 %(95% CI 34.5–72.4%) with haploidentical engraftment, respectively. (*p<0.05*) No relapse and death from relapse were recorded in patients engrafted with umbilical cord blood, but RI and RM were 25.0% (95% CI 3.2–41.9%) and 20.8% (95% CI 0–37.3%) in patients with haploidentical engraftment. NRM in recipients with haploidentical engraftment and UCB engraftment was 29.3% (95% CI 9.9–45.5%) and 15% (95% CI 0–29.3%), respectively (*p*=0.21) (**Figure 5**).

## Discussion

UCBT and haplo-HSCT have made great progress in allogeneic transplantation for patients without matched related or unrelated donors. We designed a haplo-cord HSCT protocol with modified GIAC for “the competitive transplantation of haplo-cord”. Following are the characteristics in our protocol: (1) Use low dose of ATG (total dose 5 mg/kg) for 2 days, which was the only half dose of ATG in GIAC protocol. (2) Cyclophosphamide was added after haplo HSCT on day +3, +4 for GVHD prophylaxis. (3) Peripheral stem cells were transfused on day 0, and one unit UCB was transfused on day +6. We hypothesized that the sequential transplantation of haplo-cord with the integration of ATG and PTCY for patients with hematologic malignancies could increase GVL and decrease GVHD. To verify this hypothesis, we conducted a prospectively clinical trial to compare the therapeutic effects between haplo-cord HSCT and UCB HSCT.

To our knowledge, this is the first clinical trial to compare haplo-cord HSCT with single umbilical cord blood transplantation in the setting of the myeloablative condition. Updated Eurocord guidelines suggest minimum CD34^+^ for successful engraft should be at least CD34^+^ cell dose of 1.5 × 10^5^/kg for a single unit graft (23). The CD34^+^ cells transfused less than 1.7×10^5^/kg in a single UCBT have been associated with poor engraftment, high NRM, and poor survival (24). Therefore, the combined infusion of CD34^+^ cells from a partially human leukocyte antigen (HLA)–matched UCB unit and haploid peripheral blood was a practical strategy to achieve this cell dose threshold. The haploidentical graft serves as an early myeloid bridge for the recipient to support the immune system, which provides the rapid initial granulocyte and platelet recovery. This role of “myeloid bridge” was observed and confirmed for cord engraftment in patients receiving haplo-cord HSCT (25, 26). There was a similar result in our study; myeloid engraftment occurred at a median of 14 days for patients with haplo-cord HSCT that was earlier than the median of 17 days in single cord HSCT recipients. For platelet engraftment, the median time to engraftment of 17 days significantly sooner in haplo-cord group compared with the single cord group. The results might be superior to other studies of cord blood transplant supported by third-party CD34^+^ cells, in which the median time to platelet recovery was 25–35 days (25, 27).

In our clinical trial, we used the same myeloablative regimen (Fludarabine, Cytarabine, Cyclophosphamide, and Busulfan) for both Haplo-cord and single cord HSCT groups, and both groups had no significant difference in survival outcomes at 2 years. However, in the subgroup analysis for the patients with relapsed/refractory, the 2-year probability of DFS, OS, GRFS, and NRM in haplo-cord HSCT group all showed superior outcomes. The reason might be that the immune killer cells from haploidentical and UCB donors effectively targeted remained leukemia cells in recipients with relapsed and refractory. Professor Wang’s team used the regimen of haploidentical HSCT plus infusion of UCB support for patients with relapsed/refractory. Their patients received either a busulfan (Bu)-based or total body irradiation (TBI)-based myeloablative conditioning regimen. The predominant haploidentical donor chimerism was stable achieved, which the results of 2-year OS, PFS, RI, and NRM were 35.5 and 35.5%, 25.9 and 38.0%, respectively (12). Their results showed that the haplo-cord regimen has less relapse than haplo-HSCT. Similarly, our strategy to combine transplantation of haploidentical and unrelated cord stem cells also showed better therapeutic effects than single cord HSCT for patients with relapsed/refractory. However, the conclusion should be explained cautiously due to the small case number. It is also interesting to follow up patients transplanted with our protocol for a longer time to observe the difference in survival outcomes.

Regarding the GVG to reduce relapse from two transplanted grafts such as double CBT, with the competitive effect between dUCBT, Gutman JA et al. believe that the IFN-γ secreting effector T cells from dominant naïve T cells eliminate no dominant CB cells and kill malignant cells as well (28). In addition, Lamers et al. found that the predominant UCB allele-specific effector CD4^+^ T cells induce GVG alloreactivity to eliminate recipient’s malignant cells that shared the same HLA class II alleles with non-engrafted UCB (29). It is logistic for us to speculate that haplo-cord HSCT also plays a similar GVG effect as dUCB transplantation. But the mechanism of this result in our haplo-cord HSCT recipients needs to be further investigated.

Whether GVG response increases the risk of GVHD remains controversial (12, 13, 30). In our study, the incidences of II-IV aGVHD in haplo-cord HSCT were higher than that of single cord HSCT, which was 35.4 *vs.* 12.3%, respectively (*p<0.05*), but there was no significant difference of III-IV aGVHD and cGVHD in both groups. Our results also showed that the rate of GVHD greatly reduced, and the haplo-cord HSCT had no significant difference with single cord HSCT in moderate and severe GVHD. Our result was consistent with Margaret’s one that the incidence of grade II-IV acute GVHD in dUCBT recipients was higher than that in single UCBT recipients, but not grade III-IV aGVHD (31). Besien et al. showed that cumulative incidence of grade II-IV acute GVHD was 16% after haplo-cord HSCT and 33% after haplo-HSCT (*p <*0.0001), but grade III-IV GVHD was similar (25). Kwon et al. had similar results on treating AML. In addition, the integration of low-dose ATG and PTCY regimens contributed to the decrease of GVHD (32). Salas et al. found that the use of T-cell dual depletion with ATG and PTCY for peripheral blood reduced-intensity conditioning regimen in 270 patients with allo-HSCT resulted in 4.6% acute grade III-IV GVHD and 12.4% chronic moderate/severe GVHD (33). Professor Song’s study from China also showed that using low-dose ATG and PTCY for the transplantation of Haplo-cord could effectively reduce the risk of GVHD as compared with standard-dose ATG (34), although the precondition and the time of two grafts infusion in our protocol were different from Prof. Song’s one.

Interestingly, compared to patients engrafted with haploidentical stem cells, our data revealed that the patients engrafted with umbilical cord stem cells from haplo-cord transplantation have better outcomes with a 2-year probability on OS, DFS, and GRFS, and the incidence of relapse was reduced greatly. Some reports (25, 35) had a similar result with our study. Taken together with other reports, our results indicated that cord chimerism from haplo-cord transplantation prevents disease recurrence, which might be associated with higher T-cell diversity from donors (36). Please note that although almost the same ratio of haploid or UCB chimerism (58.3 *vs.* 41.7%) has been observed in our study, we prefer to see UCB chimerism because patients with UCB engraftments were associated with better outcomes. Other studies also showed that cord chimerism prevents disease recurrence and improves progression-free survival (12, 35, 37). Many reports showed that the type of engraftment was heavily influenced by donor-recipient HLA matching level, stem cell dose, and T cells from the donor. The total nucleated cell (TNC) dose/kg (>3.0 × 10^7^/kg) and CD34^+^ cells dose/kg (3–5×10^5^ CD34^+^cells/kg) in cord blood were important for successful engraftment for UCBT (24, 38–41). Therefore, which one is determining factor of complete chimerism for engraft should be further investigated in our center. We didn’t find the meaningful key factors that determined the type of engraftment. While UCB cells were transplanted on day +6, 48 h after using CTX, avoided being damaged from CTX, which provided the chance for UCB T cells to kill T cells from haplo-HSCT. Thus, we speculated this strategy might increase the chance of stem cells from UCB for chimerism. Further study on what factors determine UCB engraftment in our patients is going on.

This study has some limitations. Firstly, multicenter clinical trials with large sample sizes and longer time could be considered to validate the results in different transplant centers. Secondly, our data showed that the survival outcomes were related to the type of haploidentical or umbilical cord blood engraftment. The mechanism on which type of engraftment dominates in recipient should be further investigated.

In conclusion, the competitive transplantation of haplo-cord improved the survival outcomes for hematological malignancies, especially for patients with relapsed/refractory. Lower-grade III-IV acute GVHD and decreased extensive chronic GVHD contributed to the overall survival outcomes after haplo-cord HSCT. Our encouraging results suggest that this promising approach is worth investigating.

## Data Availability Statement

The original contributions presented in the study are included in the article/**Supplementary Material**. Further inquiries can be directed to the corresponding authors.

## Ethics Statement

The study protocol was approved by the ethics committee of Union Hospital, Fujian Medical University. Written informed consent to participate in this study was provided by the participants’ legal guardian/next of kin.

## Author Contributions

YZC and NL conceived and designed the trial. HL and XL contributed to the analysis of the results and wrote the manuscript. YLC and DL collected data and performed the statistical analysis. PC contributed to the analysis and interpretation of the data. The remaining authors were involved in patient inclusion and data acquisition. All authors contributed to the article and approved the submitted version.

## Funding

This work was supported by National Natural Science Foundation (NSFC Grant nos. 81270641; 81541024; 81200400), Department of Science and Technology of Fujian Province Project (2017I0004 and 2017Y9057), and Top-Notch Innovative Talents Project and Fujian Project (Grants 2016Y9025 and 2016J06018). This work was also sponsored by the Construction Project of Fujian Medical Center of Hematology (Min201704) National and Fujian Provincial Key Clinical Specialty Discipline Construction Program, P. R. C. The work is part funded by Clinical Research Center for hematological malignancies of Fujian province.

## Conflict of Interest

The authors declare that the research was conducted in the absence of any commercial or financial relationships that could be construed as a potential conflict of interest.

## Publisher’s Note

All claims expressed in this article are solely those of the authors and do not necessarily represent those of their affiliated organizations, or those of the publisher, the editors and the reviewers. Any product that may be evaluated in this article, or claim that may be made by its manufacturer, is not guaranteed or endorsed by the publisher.

## Supplementary Material

Supplementary Figure S1 Distribution of the estimated propensity score in the haplo+cord and single cord group.

Supplementary Figure S2 Distribution of the estimated propensity score in the haplo+cord and single cord group.

Supplementary Figure S3 Standardized mean differences in weighted and unweighted observations.
