# Supplementary figures and images for: Sequential Transplantation of Haploidentical Stem Cell and Unrelated Cord Blood With Using ATG/PTCY Increases Survival of Relapsed/Refractory Hematologic Malignancies
Source: Front Immunol. 2021 Nov 4;12:733326. doi: 10.3389/fimmu.2021.733326 (PMC8599442; doi:10.3389/fimmu.2021.733326)

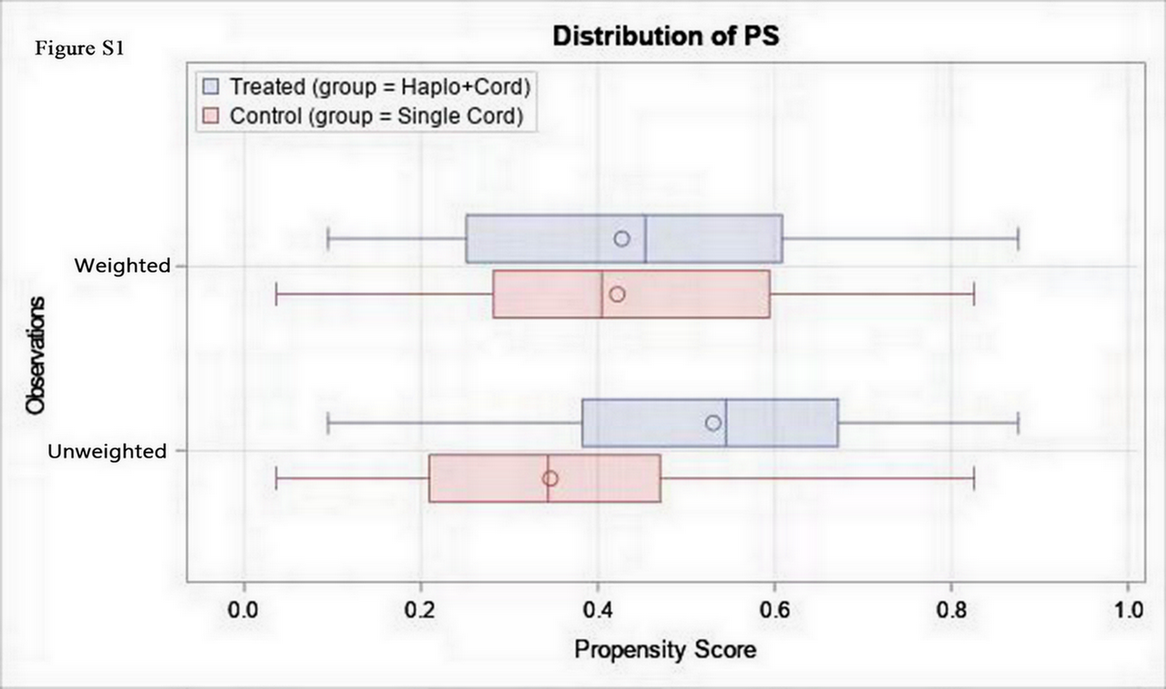

Supplement: Supplementary Figure S1 — Distribution of the estimated propensity score in the haplo+cord and single cord group. [file Image_1.tif]

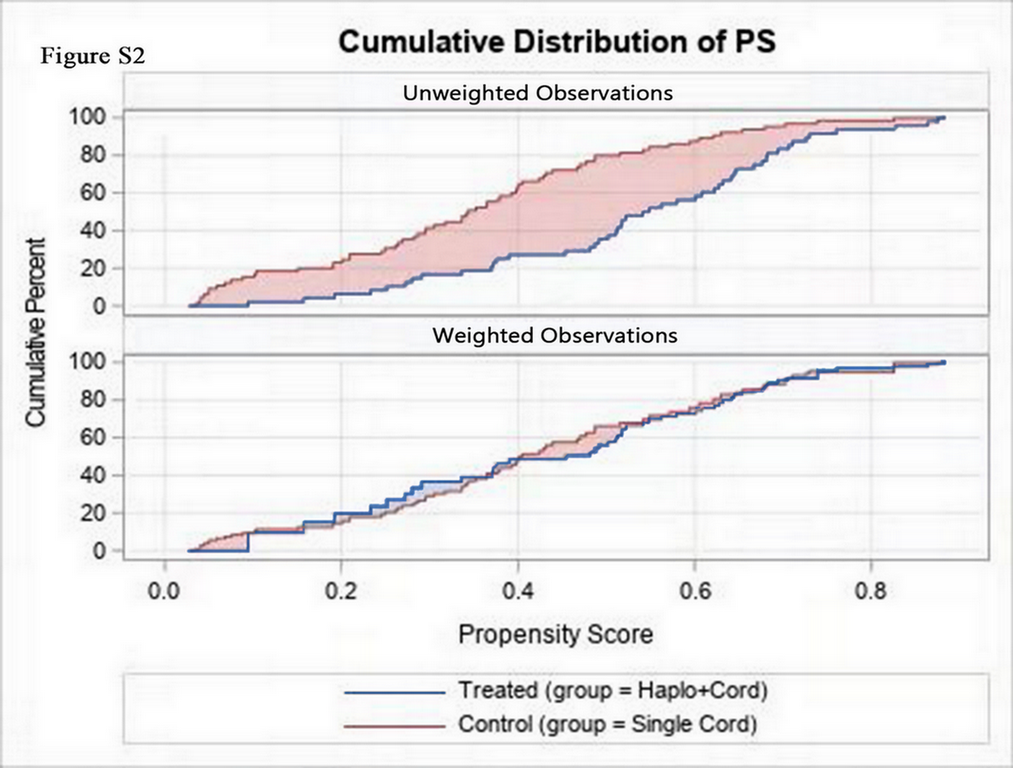

Supplement: Supplementary Figure S2 — Distribution of the estimated propensity score in the haplo+cord and single cord group. [file Image_2.tif]

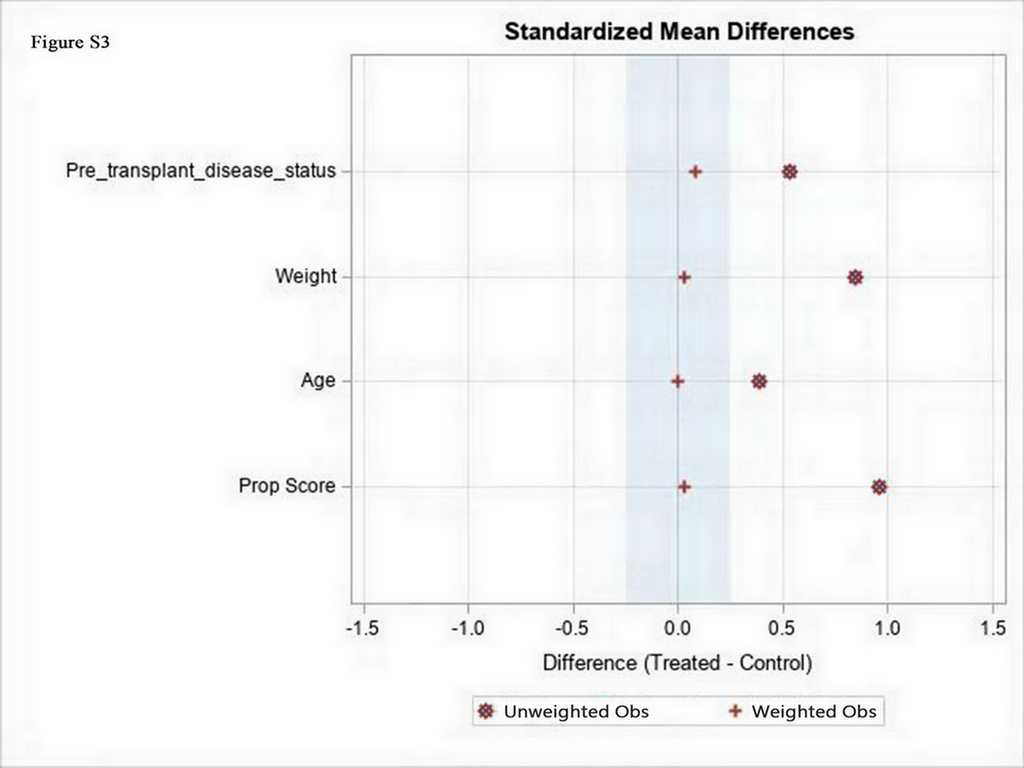

Supplement: Supplementary Figure S3 — Standardized mean differences in weighted and unweighted observations. [file Image_3.tif]
