# Supplementary material for: Sequential Transplantation of Haploidentical Stem Cell and Unrelated Cord Blood With Using ATG/PTCY Increases Survival of Relapsed/Refractory Hematologic Malignancies
Source: Front Immunol. 2021 Nov 4;12:733326. doi: 10.3389/fimmu.2021.733326 (PMC8599442; doi:10.3389/fimmu.2021.733326)
Supplement: Supplementary file 4 [file Table_1.pdf]

Table S1. The overall survival outcomes of patients received haplo+cord HSCT and single cord HSCT before and after IPTW

	unweighted			weighted		
	Haplo+cord	Single cord		Haplo+cord	Single cord	
	probabilities at 2 year % 95% CI		*p*	probabilities at 2 year % 95% CI		*p*
OS	68.0(54.5-81.5)	65.9(54.4-77.5)	0.8412	67.3 (52.0-82.5)	61.6(48.8-74.3)	0.6041
DFS	66.1(52.6-79.6)	59.9(47.9-71.9)	0.5391	65.2(49.9-80.6)	56.6(43.6-69.6)	0.4165
GRFS	62.5(48.8-76.2)	55.3(43.2-67.4)	0.5213	61.8(46.4-77.3)	49.1(36.7-61.5)	0.2945
RI	13.1(2.3-23.8)	21.3(10.5-32.2)	0.2790	11.7(0.3-23.2)	22.8(10.7-34.9)	0.1867
RM	11.4(0-22.1)	14.3(5.0-23.5)	0.5725	9.6(0-20.8)	16.2(5.7-26.7)	0.3101
NRM	23.3(11.2-35.4)	23.1(12.4-33.8)	0.8841	25.6 (11.4-39.7)	26.5(14.4-38.6)	0.9429
