# Supplementary material for: Sequential Transplantation of Haploidentical Stem Cell and Unrelated Cord Blood With Using ATG/PTCY Increases Survival of Relapsed/Refractory Hematologic Malignancies
Source: Front Immunol. 2021 Nov 4;12:733326. doi: 10.3389/fimmu.2021.733326 (PMC8599442; doi:10.3389/fimmu.2021.733326)
Supplement: Supplementary file 5 [file Table_2.pdf]

Table S2 Causes of death in haplo+cord and single cord HSCT group

Cause of death n（%）	Haplo+cord HSCT n=48	single cord HSCT n=65
Replase	4（26.6）	8（38.1）
Infection	6（40.0）	8（38.1）
Graft-versus-host disease	3（20.0）	1（4.8）
Cerebral stroke	1（6.7）	2（9.5）
Organ failure	1（6.7）	1（4.8）
Commit suicide	0	1（4.8）
Total	15	21
