# Supplementary material for: Sequential Transplantation of Haploidentical Stem Cell and Unrelated Cord Blood With Using ATG/PTCY Increases Survival of Relapsed/Refractory Hematologic Malignancies
Source: Front Immunol. 2021 Nov 4;12:733326. doi: 10.3389/fimmu.2021.733326 (PMC8599442; doi:10.3389/fimmu.2021.733326)
Supplement: Supplementary file 6 [file Table_3.pdf]

Table S3 *p* value of Cox regression Analysis

**Outcomes**	Group	Pre-transplant disease status	The interaction between Group and Pre-transplant disease status
**OS**	0.0027	<.0001	0.0064
**DFS**	0.002	<.0001	0.0082
**GRFS**	<.0001	<.0001	0.0006
**RI**	0.3655	0.325	<.0001
**NRM**	0.0601	0.001	0.0265
**RM**	0.1848	0.0303	0.8019
