# Supplementary material for: Sequential Transplantation of Haploidentical Stem Cell and Unrelated Cord Blood With Using ATG/PTCY Increases Survival of Relapsed/Refractory Hematologic Malignancies
Source: Front Immunol. 2021 Nov 4;12:733326. doi: 10.3389/fimmu.2021.733326 (PMC8599442; doi:10.3389/fimmu.2021.733326)
Supplement: Supplementary file 7 [file Table_4.pdf]

Table S4. The survival outcomes of patients with CR (MRD-positive or MRD-negative) between groups.

outcomes	Group	unweighted				weighted			
		**MRD negative**		**MRD positive**		**MRD negative**		**MRD positive**	
		HR (95%CI)	*p*	HR (95%CI)	*p*	HR (95%CI)	*p*	HR (95%CI)	*p*
OS	Single cord								
	Haplo+cord	1.178(0.345, 4.024)	0.7943	1.421(0.316, 6.394	0.6468	1.909(0.855, 4.262)	0.1145	0.946(0.343, 2.61)	0.915
DFS	Single cord								
	Haplo+cord	0.918(0.319, 2.641)	0.8733	1.259(0.281, 5.636	0.7630	1.378(0.701, 2.709)	0.3522	0.816(0.296, 2.252)	0.695
GRFS	Single cord								
	Haplo+cord	0.935(0.355, 2.459)	0.8910	1.259(0.281, 5.636	0.7630	1.447(0.779, 2.687)	0.2418	0.816(0.296, 2.252)	0.695
RI	Single cord								
	Haplo+cord	0.947(0.180, 4.976)	0.9491	NA	NA	0.751(0.225, 2.501)	0.6403	NA	NA
NRM	Single cord								
	Haplo+cord	1.248(0.300, 5.196)	0.7607	1.638(0.264, 10.15[illegible]	0.5960	2.287(0.972, 5.382)	0.0582	1.142(0.367, 3.554)	0.818
RM	Single cord								
	Haplo+cord	[illegible].003(0.089, 11.288	0.9983	0.753(0.083, 6.818	0.8007	0.756(0.112, 5.117)	0.7745	0.756(0.112, 5.117)	0.775
