# Supplementary material for: Sequential Transplantation of Haploidentical Stem Cell and Unrelated Cord Blood With Using ATG/PTCY Increases Survival of Relapsed/Refractory Hematologic Malignancies
Source: Front Immunol. 2021 Nov 4;12:733326. doi: 10.3389/fimmu.2021.733326 (PMC8599442; doi:10.3389/fimmu.2021.733326)
Supplement: Supplementary file 8 [file Table_5.pdf]

Table S5. The baseline characteristics of intention-to-treat population in haplo+cord and single cord group

Characteristics	unweighted population		
	Haplo+Cord	Single Cord	P value
**Total patients**	32	38	
**Median age at diagnosis, years (range)**	28 (6-56)	23(1-46)	0.0285
**Weight(range)**	65(20.1-90.0)	51(1.01-80.0)	0.0005
**Median follow-up months (range)**	21.6（1.5-49.6）	36.2（0.1-71.9）	/
**Patient gender**（n，%）			0.0292
Male	21（65.6）	15(39.5)	
Female	11（34.4）	23(60.5)	
**Diagnosis（n，%）**			0.6026
AML	14（43.8）	15（39.5）	
ALL	16（50）	22（57.9）	
MDS	1（3.1）	0（0）	
CML-AP	1（3.1）	1（2.6）	
**HCT-CI score n (%)**			0.9019
0-1	31（97.9）	37 (97.4)	
2	1（3.1）	1 (2.6)	
**Interval diagnosis to transplant，n (%)**			0.9001
<12 months	24（75.0）	28（73.7）	
>=12months	8（25.0）	10（26.3）	
**Disease status at transplant, n (%)**			0.2575
First CR	17（53.1）	24（63.2）	
Second CR or greater	2（6.3）	5（13.1）	
Refractory/ Relapse	13（40.6）	9（23.7）	
**MRD status with CR at transplant，n (%)**			0.8334
MRD positive	4(21.1)	4(14.3)	
MRD negative	15(78.9)	24(85.7)	
